# Feasibility, acceptability and potential effectiveness of dignity therapy for family carers of people with motor neurone disease

**DOI:** 10.1186/1472-684X-13-12

**Published:** 2014-03-19

**Authors:** Brenda Bentley, Moira O’Connor, Lauren J Breen, Robert Kane

**Affiliations:** 1 School of Psychology and Speech Pathology, Faculty of Health Sciences, Curtin University, GPO Box U1987, Perth, WA6845, Australia

**Keywords:** Motor neurone disease, Amyotrophic lateral sclerosis, Palliative care, Family carers, Dignity therapy, Psychotherapy, Family therapy

## Abstract

**Background:**

Dignity therapy is a brief psychotherapy that has been shown to enhance the end of life experience. Dignity therapy often involves family carers to support patients weakened by illness and family carers are also the usual recipients of the legacy documents created. No research to date has examined the impact of dignity therapy on family carers at the time of the intervention. This study examined the effects of dignity therapy on family carers of people with motor neurone disease (MND).

**Methods:**

This is a cross-sectional study utilizing a one-group pre-test post-test design with 18 family carers of people diagnosed with MND. Outcomes measured caregiver burden, anxiety, depression, and hopefulness. Acceptability was measured with a questionnaire. Feasibility was assessed by examining family carers’ involvement in the therapy sessions, time taken to conduct sessions, and any special accommodations or deviations from the dignity therapy protocol.

**Results:**

There were no significant pre-test post-test changes on the group level, but there were decreases in anxiety and depression on the individual level. Baseline measures indicate that 50% of family carers had moderate to severe scores for anxiety prior to dignity therapy. MND family carers saw benefits to the person with MND and to themselves after bereavement, but acceptability of dignity therapy at the time of the intervention was mixed with some family carers indicating it was helpful, some indicating it was harmful, and many expressing ambivalence. Dignity therapy involving MND family carers is feasible and the involvement of family carers has minimal impact on the therapy.

**Conclusion:**

Dignity therapy is not likely to alleviate caregiver burden in MND family carers, but it may have the ability to decrease or moderate anxiety and depression in distressed MND family carers. Dignity therapy is feasible and generally acceptable to MND family carers. Dignity therapists may provide a better experience for family carers when they are aware of acceptance levels and the quality of partner relationships.

**Trial registration:**

ANZCTR Trial Number: ACTRN12611000410954

## Background

Dignity therapy is a brief psychotherapeutic intervention which has been shown to enhance the end of life experience in people with life-limiting conditions [1]. In a distressed group of terminally ill patients, dignity therapy has been shown to reduce anxiety and depression [2]. Dignity therapy is based on a theoretical model of dignity in the terminally ill [3] and its purpose is to reduce dignity-related distress while enhancing hope and meaning [4]. Dignity therapy offers people who are facing death an opportunity to create a legacy document. In a recorded interview, guided by a therapist, a person is invited to recount aspects of his/her life to be remembered, discover purpose and meaning in life, and express final words or advice to loved ones. The therapist and client work together to edit the interview transcript, and the therapy concludes when a final document is produced which can be shared with family and friends [5].

Dignity therapy often involves family carers who may provide support during the therapy interview (s) and editing process, and who may also assist a family member weakened by illness in providing the narrative [6]. Family members are also the usual recipients of the documents, making them an important part of the therapy even when they are not involved in the document’s creation. Despite the importance of family members’ involvement in dignity therapy, no studies to date have examined the impact of the therapy on family members at the time of the intervention, though dignity therapy has been shown in prior research to moderate the bereavement experience of family members after their loved one had died [7].

This study examined the effects of dignity therapy on the family carers of people with motor neurone disease (MND). A person with MND endures progressive paralysis and gradually loses the abilities to move, speak, swallow and breathe. There is no treatment or cure. Median life expectancy is 2–4 years after diagnosis and death is most often caused by respiratory failure [8]. Family members caring for people with MND often encounter exceptional strain during the caregiving experience due to the rapid and progressive nature of MND coupled with the incapacitating effects of the disease. Research has documented the distress and burden experienced by MND family carers [9-12], and shown the close correlation of distress levels in patient-carer couples [13,14]. Moreover, carer burden increases in MND family carers over time as patient function declines [15-17].

Quality of life may be increased and perceived carer burden decreased in MND family carers who find positive meaning [13,14] and have a sense of hope [18].

Dignity therapy is an intervention designed to bolster hope and meaning and could therefore alleviate perceived burden. Dignity therapy also shows promise to enhance the end of life for people with MND, but its impact on carer distress and burden should be considered when evaluating its overall impact.

### Aims and objectives

The aim of this study was to assess the feasibility, acceptability, and potential effectiveness of dignity therapy for family carers of people with MND. The specific objectives were to assess the impact of dignity therapy on family carers at the time of the intervention by determining whether:

a) dignity therapy decreases perceived caregiver burden, anxiety, depression, and increases hope in MND family carers;

b) dignity therapy is acceptable to MND family carers; and

c) it is feasible to involve MND family carers in the delivery of dignity therapy.

## Methods

### Study design

This cross-sectional study utilized a single treatment group and a pre-test post-test design. A control group was not utilized due to 1) the need to test the feasibility of dignity therapy with people with MND because they are a new research population for this intervention [19]; 2) access issues to the small MND population in Western Australia; and 3) ethical concerns over making a potentially useful intervention unavailable to a control group. More information about the design is available in the study protocol [20].

### Ethical approval

This study was approved by the Curtin University Human Research Ethics Committee (19/2011).

### Setting

MND family carers were recruited from Western Australia (WA) as a result of outreach from the Motor Neurone Disease Association of WA (MNDAWA). MNDAWA provides services to 100–120 diagnosed people with MND at any one time.

### Participants

Family carers of adults diagnosed with MND who could communicate in English were eligible for the study. Enrolment occurred between June 2011 and July 2013. Family carers were invited to participate in the study when a person with MND enrolled to complete dignity therapy as a part of a larger research study. If the family carer did not elect to participate, or if the participant did not have a family carer, the person with MND remained eligible to continue with the study. People with MND and their family carers were excluded if the person with MND could not provide informed consent (based on the ALS-Cognitive Behavioural Screen [21] and/or the Blessed Orientation Memory Concentration Test (BOMC) [22]), were too ill to complete dignity therapy, or were unable to communicate in English. There was no selection criteria based on distress levels, stage of disease or proximity to death.

### The intervention and study procedures

The intervention was administered by the first author as part of her PhD studies, who was trained in dignity therapy by Harvey Max Chochinov who developed dignity therapy [1,4]. The therapy interviews were audio-recorded and transcribed verbatim. Consistent with the dignity therapy protocol, some family carers provided support and assisted with the interview at the request of the person with MND [5]. The researcher shaped the transcribed interviews using the prescribed editing process [5]. The legacy document was finalised with the aid of the person with MND and, where relevant, his or her family carer. The researcher read the document to each person with MND and to family members who wished to attend the reading. Post-testing occurred via mail or a visit from a second researcher to mitigate response bias. To assure adherence to the dignity therapy protocol, the researcher engaged in regular supervision from Prof. Chochinov and three experienced researchers (two trained in dignity therapy) reviewed three recordings, transcripts and completed documents (10%), which were deemed to be adherent.

### Measures and outcomes

#### Effectiveness

Outcome data to measure potential effectiveness were collected from family carers at baseline and one week after completion of dignity therapy. The primary outcome is the family carers’ sense of perceived **burden,** measured by the Zarit Burden Inventory [23], a reliable (α = 0.87) validated instrument with a summative score ranging from 0–48 where higher scores indicate more burden. Secondary outcomes were: 1) **hopefulness** assessed with the Herth Hope Index [24,25], a reliable (α = 0.97) validated instrument developed for people confronting terminal illness and their families with a score ranging from 12–48 and where higher scores indicate more hopefulness; 2) **anxiety** and 3) **depression,** measured with the Hospital Anxiety and Depression Scale (HADS) [26], an instrument often used with family caregivers showing strong reliability (α = 0.89) and validity. HADS scores range from 0–21 with scores of 8–10 indicating moderate distress and 11–21 indicating severe distress on both the anxiety and depression sub-scales.

#### Acceptability

A family feedback questionnaire was used to collect family carers’ opinions and experiences of the intervention. The questionnaire contained 20 questions answered with a 5-point Likert scale and space for brief explanation.

#### Feasibility

Data were collected about the family carers’ involvement in the therapy sessions, time taken to conduct the dignity therapy sessions, any special accommodations made in the delivery of the intervention when family carers were involved, deviations from the dignity therapy protocol, reasons for non-completion and reasons for attrition.

#### Demographic and health status

Level of impairment of the person with MND and change in physical function over time was collected from the family carer using the Amyotrophic Lateral Sclerosis Functional Rating Scale-R (ALS-FRS) where scores range from 0–48 (lower scores indicating more impairment) [27,28]. Possible cognitive behavioural impairment of the person with MND was assessed with the ALS-CBS, which contains a questionnaire for the person with MND and a separate questionnaire for the family carer [21]. Demographic data on age, gender, relationship to the person with MND, children in the home, caring hours per day, support received, employment status, spirituality, and health history were also collected.

### Analysis

Data were analysed with generalized mixed models (GLMM) as implemented through SPSS’s (Version 20) GENLINMIXED procedure. Model parameters were estimated with robust standard errors to accommodate potential violations of the model assumptions. Participant was treated as a random effect and Time (pre-test, post-test) was treated as a fixed effect. Caregiver age, gender, level of education, employment status, spirituality, relationship length, and caring hours were treated as fixed effects and analysed individually as potential moderators of the intervention effect. In order to optimise the likelihood of convergence, a separate GLMM analysis was run for each of the four outcome measures. The GLMM maximum likelihood procedure is a full information estimation procedure that uses all data present at each assessment point, which reduced sampling bias associated with participant attrition. GPower (Version 3.1) indicated that 18 participants would be sufficient to capture relatively 'large’ (*f* = .36) pre-post changes on the outcome variables. Descriptive statistics were used to summarize demographic variables and feedback responses.

## Results

### Response rate

We recruited 18 family carers from the study group of 29 people with MND. Six people with MND had family carers who were unwilling or unable to participate (family carer response rate 75%). The reasons for non-participation were either the person with MND did not wish to ask their partner to participate (*n* = 3), or the family carer declined stating they did not have the time (*n* = 3). Five people with MND had no family carers. All 18 family carers completed the study, though one returned only the feedback questionnaire and not the post-test measures.

### Demographic information

Family carers (13 women, 5 men) were aged from 38 to 80 years with a median age of 61. All 18 were spouses/partners who resided with the person with MND. See Table 1 for more demographic information on our study group.

**Table 1:** Demographic characteristics of the study group

**Characteristic**	***N***
**Gender**	
Male	5
Female	13
**Age**	
30-39	2
40-49	2
50-59	3
60-69	7
70-79	3
80-89	1
**Relationship to person with MND**	
Spouse/partner	18
**Length of relationship to person with MND**	
5 to 10 years	1
10 to 25 years	3
More than 25 years	14
**Residence area**	
Urban/metropolitan	14
Rural	4
**Highest level of education attained**	
Primary/elementary school	1
Secondary/high school	11
University/technical	5
Postgraduate	1
**Number of minor children living at home**	
0	15
1	2
3	1
**Current employment status**	
None	13
Full-time	3
Part-time	2
**Caring hours per day**	
Less than 4 hours	5
4 to 8 hours	2
8 to 12 hours	1
More than 12 hours	10
**Time since MND diagnosis of family member**	
Less than one year	4
One to two years	9
Two to three years	2
Three to four years	0
More than four years	3
**Are you currently being treated for a major medical condition?**	
No	6
Yes	12
**Have you been prescribed medication to help you cope?**	
Anti-depressant	1
Anti-anxiety	1
No	16
**Do you consider yourself to be a spiritual person?**	
Yes	3
Somewhat	10
No	5
**Cognitive screening scores of person with MND**	
No impairment	11
Suspected mild to moderate impairment	7
**Formal support received to help cope with family member’s MND**^**a**^	
None	7
Support group	3
Home care	4
Respite	1
Counselling	1
Psychologist/Psychiatrist	2
Other:	
MND Association	3
Church	1
Massage	1
Medication	1

*Note.*^*a*^*Participants could list more than one type of support.*

### Baseline levels of impairment and distress for clients and carers

People with MND who were cared for by family carers had low to moderate physical impairment (mean ALS-FRS score was 32.61). Seven carers cared for family members who were mildly to moderately cognitively impaired as measured by the ALS-CBS. The carers reported moderate baseline levels of distress. Half of the family carers had moderate (*n* = 6) to severe (*n* = 3) scores for anxiety. Depression was less common with three carers reporting moderate scores for depression.

### Effectiveness

Family carers reported a significant pre-post increase in burden in conjunction with a significant pre-post decrease in the physical functioning of the patient. After controlling for the pre-post decrease in physical functioning of the person with MND, the pre-post increase in the carer burden was no longer significant (*F* [1,32] = 3.32, *p* = .078, *d* = .30). There were no significant pre-post changes in self-reported hopefulness, anxiety, or depression. Pre-test and post-test descriptive statistics and test statistics for pre-post differences are reported for all outcome variables in Table 2.

**Table 2:** Mean pre-test post-test scores (and standard deviations) for measures of burden, hopefulness, anxiety, depression, and physical function

**Outcome**	**Pre-test *N*** **= 18**	**Post-test *N*** **= 17**	***F *** **(1.33)**	***P***	***d***
Caregiver burden (ZBI)	12.44 (7.89)	16.29(11.22)	5.58	.024	0.95
Hopefulness (HHI)	38.39 (4.46)	36.71 (4.52)	3.19	.083	0.62
Anxiety (HADS)	7.28 (3.71)	6.88 (4.33)	1.33	.257	0.26
Depression (HADS)	4.17 (3.33)	4.41 (3.91)	0.03	.860	0.39
Physical function (ALS-FRS)	32.61 (9.76)	30.12 (9.62)	7.00	.012	1.19

Potential moderators of the intervention effect (caregiver age, gender, level of education, employment status, spiritual beliefs, how long carers had known the patient, caring hours per day, and the number of children living at home) were individually entered in the regression model in order to determine whether positive pre-post changes would emerge at certain levels of the moderator. There was no significant Moderator x Time interactions for any outcomes (all *p*s > .1).

A reliable change (RC) score for each carer [29] was computed to investigate the presence of reliable pre-post change at the individual rather than group level. The RC score is the degree to which the person changes on the outcome variable divided by the standard error of difference between the pre- and post-test scores. When the absolute value of the RC score is greater than 1.96, (Wise [30] has argued that this value can be reduced in some situations), it is likely that the post-test score reflects a *real* or *reliable* change. The results suggest potential prevention effects for anxiety and depression, and both increases and decreases in hopefulness (see Table 3).

**Table 3:** Number of carers showing reliable improvement, deterioration, and no change for burden, hopefulness, anxiety, and depression

**Outcome**	**Improved**	**Deteriorated**	**No change**	***N***
Caregiver burden (ZBI)	0	4	13	17
Hopefulness (HHI)	3	8	6	17
Anxiety (HADS)	2	0	15	17
Depression (HADS)	1	1	15	17

### Acceptability

The reported benefits and acceptability of dignity therapy to the family carers of people with MND was mixed. There were many feedback responses indicating it was helpful, some indicating it was not helpful or even harmful, and some showing ambivalence. Half (*n* = 9) of the family carers agreed or strongly agreed that dignity therapy was helpful to them, while about a quarter (*n* = 4) disagreed or strongly disagreed. More family carers disagreed than agreed that dignity therapy helped to reduce their feelings of stress as a carer (*n* = 5 v *n* = 6) and an equal numbers agreed as disagreed that it helped them feel closer to their partner (*n* = 6 v *n* = 6). Some family carers appeared to find dignity therapy confronting and mentioned the attention it brought to their partners’ impending death.

Family carers were more positive about seeing benefits to their partner, with 16 agreeing or strongly agreeing that dignity therapy had been helpful to their family member, 11 reporting it was an important component of their care, 10 reporting it had increased meaning in their partner’s life, and 9 indicating it had helped their family member prepare for the end of life. Despite mixed feelings about the therapy, 13 believe the document would be an ongoing source of comfort and 14 would recommend dignity therapy to other people with MND and their families. The complete results of the feedback questionnaire are reported in Table 4. Selected comments are available in Table 5.

**Table 4:** Results of the Family Feedback Questionnaire

**Item**	**Family carers (*N*** **= 18)**			
	**Mean**	**SD**	***n *** **Agree (SA + A)**	***n *** **Disagree (SD + D)**
**DT was helpful to my family member**	4.22	0.647	16	0
**DT has given my family member a heightened sense of purpose or meaning**	3.87	1.060	10	2
**DT helped increase my family member’s sense of dignity**	3.56	0.984	8	2
**DT helped prepare my family member for the end of life, whenever that may occur**	3.33	0.970	9	3
**DT was an important component of my family member’s care as any other aspect of their care, including symptom management**	3.61	0.979	11	3
**DT helped reduce my family member’s suffering**	3.22	1.003	7	5
**DT helped increase my family member’s hopefulness about the future**	3.17	0.857	6	4
**The DT document helped me during this time of our life**	3.33	1.085	9	4
**DT has helped me prepare for the end of life of my family member, whenever that may occur**	3.11	0.832	5	4
**DT was helpful in reducing my feelings of stress as a carer**	3.00	0.907	5	6
**DT helped me feel closer to my family member**	2.94	0.938	6	6
**DT has increased my hopefulness about the future**	3.11	0.758	6	4
**The DT document will continue to be a source of comfort for my family and me**	3.83	0.618	13	0
**I would recommend DT to other patients of family members who are dealing with MND**	4.00	0.686	14	0
**Scoring: 1 *strongly disagree*, 2 *disagree*, 3 *neither agree or disagree*, 4 *agree*, 5 *strongly agree*.**				

**Table 5:** Selected comments from the family feedback questionnaire

**Item**	**Comment**
The dignity therapy document helped me during this time of our life.	“It has provided a source of information and inspiration”.
	“I didn’t learn anything about him I didn’t already know”.
	“I put my husband first and yet what I read I didn’t feel very appreciated or loved”.
Dignity therapy was helpful in reducing my feelings of stress as a carer.	“Answering the questions actually increases the stress”.
	“When the real suffering begins the stress is going to come no matter what”.
	“[There is] more understanding, less tension”.
Dignity therapy helped me feel closer to my family member.	“Some days we are on the same page, but other days we are upset, angry and not close at all”.
	“Nothing replaces 50+ years of constant close companionship and mutual caring”.
	“We have always been close but I feel more protective now”.
Dignity therapy has helped me prepare for the end of life of my family member, whenever that may occur.	“I don’t see how relating his life in a few short pages could prepare me”.
	“One thing it did do was focus on the end and not to live and enjoy the journey along the way the best we can”.
	“[It helped] from pushing aside the situation to more acceptance”.
Dignity therapy was helpful to my family member.	“Just for him to think of the past and what he has achieved in his life is satisfying”.
	“She expressed emotions which she normally suppresses”.
	“He enjoyed the opportunity to put memories on paper and have something concrete for others to read”.
Dignity therapy helped prepare my family member for the end of life, whenever that may occur.	“[We] recently went on a family holiday and he was able to talk to his children about his condition”.
	“He’s been in denial but has recently come to terms with his diagnosis and the dignity therapy helped through giving an opportunity to talk about these issues”.
	“It has made him face up to his situation and to express himself to family and friends”.
The dignity therapy document will continue to be a source of comfort for my family and me.	“If we are missing him, we can just read the booklet”.
	“The document will provide a basis for reference and reflection”.
	“We will treasure his story forever”.

### Feasibility

Twelve of the 18 family carers assisted with the interview and editing process. Ten family carers provided support by attending the therapy sessions and contributing to the narrative when requested by their partner, one served as a proxy for her husband who had lost the ability to speak and write (completing the entire interview in his presence with minimal contribution from her husband), and one carer of a speech impaired wife was significantly involved in providing detail and elaborating on her responses which were written on a whiteboard. However, not all speech impaired participants required or requested assistance from their family carers as several relied on assisted communication, including email, to complete the interview and editing. We analysed the data using descriptive statistics to determine if the distress levels or acceptability levels differed between the family carers involved and those not involved in the interviews and editing and we found no significant differences. The family member who served as a proxy was the only deviation from the dignity therapy protocol, though Chochinov and colleagues report instances of dignity therapy conducted via family proxies and it appears that this is an acceptable deviation [6].

Generally, individual dignity therapy meetings involving family carers (*n* = 12) were of a longer duration than those completed by the client alone (*n* = 17). Family carers added to the session dialog, asked questions, and often provided refreshments or engaged in other caring tasks that made their partner more comfortable. All of these actions extended sessions. Nonetheless, involvement of family carers did not equate to longer legacy documents. For clients assisted by family carers, documents were 7 to 47 pages (mean 20.42, *SD* 13.35), while documents were 11 to 57 pages (mean 22.94, *SD* 10.615) for clients who completed the therapy alone. The number of sessions required to complete the therapy was fewer in the group assisted by family carers (mean 3.75 v 4.41), and the days to complete the intervention slightly more in the group assisted by family carers (mean 46 v 39).

## Discussion

Dignity therapy has been shown to moderate the bereavement experience in family carers when they were interviewed 9 to 12 months after death [7], but no previous studies have looked at the impact of the therapy on family carers at the time of the intervention. We hypothesised post-intervention decreases in burden, anxiety and depression scores and an increase in hope, but there were no significant changes. Rather, our population showed an increase in burden which correlated to a decline in the physical function of the patient during the study period. This effect is consistent with research of burden over time in MND family carers [15-17]. Without a control group, we are unable to ascertain whether dignity therapy had a prevention effect against expected increases in burden, anxiety, and depression, or a decline in hopefulness.

The individual results on anxiety and depression are more encouraging and suggest that dignity therapy has the potential to decrease anxiety and depression in family carers who are experiencing moderate to high levels of distress. This is similar to the findings for terminally ill cancer patients, where distressed individuals had a decrease in anxiety and depression scores [2], but those with low baseline levels of distress showed no change [1].

Acceptability of dignity therapy was mixed. Family carers felt that the therapy provided a benefit to their family members and that the document would help them in bereavement, and most rated the experience as satisfactory and one they would recommend to others. Whether a family carer was directly involved in the therapy had little impact on the acceptability or feasibility of dignity therapy. Rather, the comments provided on the feedback questionnaire suggest that family carers’ level of acceptance of their partner’s imminent death, or the quality of the relationship between family carer and partner, may lead to dignity therapy having a potentially negative impact on family carers at the time of the intervention.

### Strengths and limitations

The strengths of this study were the high response rate and high completion rate of MND family carers, the use of MND-specific cognitive and health status measures, and the demographic characteristics of the sample are generally representative of MND family carers. The limitations include inadequate power to discover small to moderate effects, mild to moderate levels of distress at baseline, and the lack of a control group. The study group may not be representative of all MND family carers because those who declined to participate may have been more distressed, and people with MND with severe cognitive impairment and their family carers were excluded from the study.

### Implications for future research

The effectiveness of dignity therapy in decreasing perceived caregiver burden, anxiety or depression, or increasing hopefulness in MND family carers could not be determined in this study. A randomised controlled trial with a greater number of participants is needed, perhaps incorporating a stepped-wedge or cluster design. An experimental study focusing on distressed family carers is warranted to determine if dignity therapy has the potential to decrease anxiety and depression. A qualitative study with family carers is indicated to explore more fully the mixed acceptability results provided in the feedback questionnaire. Further, a longitudinal study would determine if the document was helpful to carers following bereavement.

### Ethical challenges

Dignity therapy may trigger emotional upset in people with MND who are experiencing emotional lability and this was frequently encountered. Emotional lability, also known as pseudobulbar affect, is an MND symptom which has the potential to cause distress to the person with MND and their family carer if it is not treated sensitively and therapeutically. In our study, we provided a supportive and safe environment, information and psycho-education, and normalised the symptom. Other ethical challenges encountered include negotiating complex relationships of participants and family carers, minimising harm to both parties as well as extended family members through what was written in (or left out of) the generativity document, and balancing the interests of the family carers with those of the patient. These challenges suggest that the skillful application of dignity therapy by a trained psychotherapist who is knowledgeable about MND is paramount in any future research.

## Conclusions

The findings of this study suggest that dignity therapy is not likely to alleviate the burden encountered by MND family carers during caring, but it may have the ability to decrease or moderate anxiety and depression in distressed MND family carers. Dignity therapy is feasible and generally acceptable to family carers of people with MND, who recognize benefits to their ill family member and also the potential benefits to themselves during bereavement as a result of having the legacy document. Comments from carers indicate that it is important for a dignity therapist to be aware of acceptance levels and dynamics in partner relationships in order to best provide a satisfactory experience for family carers.

## Competing interest

The authors declare that they have no competing interests.

## Authors’ contributions

BB and MO designed the study. MO and LB supervised the research. BB conducted the research and drafted the article. RK provided substantial assistance with the data analysis. MO, LB, and RK made substantial contributions to the critical revision of the article. All authors read and approved the final manuscript.

## Pre-publication history

The pre-publication history for this paper can be accessed here:

http://www.biomedcentral.com/1472-684X/13/12/prepub
