# Erratum to: Feasibility, acceptability and potential effectiveness of dignity therapy for family carers of people with motor neurone disease

**DOI:** 10.1186/s12904-016-0078-7

**Published:** 2016-02-17

**Authors:** Brenda Bentley, Moira O’Connor, Lauren J. Breen, Robert Kane

**Affiliations:** School of Psychology and Speech Pathology, Faculty of Health Sciences, Curtin University, GPO Box U 1987, Perth, WA6845 Australia

## Erratum

The acknowledgement of our article [1] published in *BMC Palliative Care* has been incorrect with respect to the contributions of Samar Aoun and Harvey Max Chochinov who also contributed to the conception and design of the project. The corrected acknowledgement is presented here.

### Acknowledgement

The authors thank Samar Aoun and Harvey Max Chochinov for their assistance with the project, for securing the funding for this study, and acknowledge their contribution to the conception and design of the project. We are grateful for the support of the Motor Neurone Disease Association of Western Australia. Finally, we wish to thank all of the people who took part in this study.

## Editorial note

The editorial department of *BMC Palliative Care* would like to inform its readers that the corresponding author has changed affiliation since the publication of the original article [1] and can now be contacted via b.bentley@murdoch.edu.au.
